# Hospital Surgical Volume and 3-Year Mortality in Severe Prognosis Cancers: A Population-Based Study Using Cancer Registry Data

**DOI:** 10.2188/jea.JE20190242

**Published:** 2021-01-05

**Authors:** Yukari Taniyama, Takahiro Tabuchi, Yuko Ohno, Toshitaka Morishima, Sumiyo Okawa, Shihoko Koyama, Isao Miyashiro

**Affiliations:** 1 Department of Mathematical Health Science, Graduate School of Medicine, Osaka University, Osaka, Japan; 2 Cancer Control Center, Osaka International Cancer Institute, Osaka, Japan

**Keywords:** 3-year mortality, biliary tract cancer, esophageal cancer, hospital surgical volume, pancreatic cancer

## Abstract

**Background:**

The impact of hospital surgical volume on long-term mortality has not been well assessed in Japan, especially for esophageal, biliary tract, and pancreatic cancer, although these three cancers need a high level of medical-technical skill. The purpose of this study was to examine associations between hospital surgical volume and 3-year mortality for these severe-prognosis cancer patients.

**Methods:**

Patients who received curative surgery for esophageal, biliary tract, and pancreatic cancers were analyzed using the Osaka Cancer Registry data from 2006–2013. Hospital surgical volume was categorized into tertiles (high/middle/low) according to the average annual number of curative surgeries per hospital for each cancer. Three-year survivals were calculated using the Kaplan-Meier method. Hazard ratios (HRs) of 3-year mortality were calculated using Cox proportional hazard models, adjusting for patient characteristics.

**Results:**

Three-year survival was higher with increased hospital surgical volume for all three cancers, but the relative importance of volume varied across sites. After adjustment for all confounding factors, HRs in middle- and low-volume hospitals were 1.34 (95% confidence interval [CI], 1.14–1.58) and 1.57 (95% CI, 1.33–1.86) for esophageal cancer; 1.39 (95% CI, 1.15–1.67) and 1.57 (95% CI, 1.30–1.89) for biliary tract cancer; 1.38 (95% CI, 1.16–1.63) and 1.90 (95% CI, 1.60–2.25) for pancreatic cancer, respectively. In particular for localized pancreatic cancer, the impact of hospital surgical volume on 3-year mortality was strong (HR 2.66; 95% CI, 1.61–4.38).

**Conclusion:**

We suggest that patients who require curative surgery for esophageal, biliary tract, and pancreatic cancer may benefit from referral to high-volume hospitals.

## INTRODUCTION

It has been argued that procedural volume number per hospital for cancer surgery is an important determinant of outcomes, such as survival and mortality.^1^ Hospital volume is an index that indicates the total technical level of medical facilities to evaluate the effect of the levels on patient outcomes.^2^^–^^6^ A high level of technical-medical skill is necessary for surgical resection, especially in severe-prognosis cancers, such as esophageal and pancreatic cancer. Previous studies have shown that the difference between high- and low-volume hospitals could be explained by the experience of the surgeons. As surgeons in high-volume hospitals have extensive experience of cancer surgery, the mortality of patients whose operations are carried out by such surgeons would decrease.^7^^–^^10^

In most previous studies examining hospital surgical volume, short-term mortality, such as in-hospital, within 30 days, and within 90 days, has been used as an outcome.^2^^,^^3^^,^^5^^,^^11^ However, long-term mortality, such as 3- and 5-year survival, would be also important, because long-term survival represents the possibility of cure in most cancer patients.

To promote cancer control in Japan, the Japanese government instigated centralization of patients and treatments to designated cancer care hospitals, which play central roles in cancer care in Japan, but focus on five major cancers: stomach, colorectal, liver, lung, and breast cancer, which are among the most common cancers in Japan.^12^

In Japan, the relationship between hospital surgical volume and 5-year mortality for stomach, breast, uterus, and ovary cancer, some of which was major cancer in Japan, was reported that long-term mortality in these major cancers was high especially in very-low volume hospitals.^13^^–^^16^ The results suggested that cancer care for major cancers were equalized in Japan. However, information on hospital volume of other cancers, such as esophageal, biliary tract, and pancreas cancers, which have severe prognoses even at early stages, is very limited in Japan.^17^ Even globally, very few studies have examined the relationship between hospital surgical volume and long-term mortality for biliary tract cancer.^18^

For esophageal, biliary tract, and pancreatic cancer patients who had surgery, prognosis was better in high-volume than in low-volume hospitals worldwide. However, adjustment factors varied in those studies, and the effect of hospital surgical volume was not defined by cancer stage.^1^^–^^6^^,^^8^^–^^11^

Thus, we aimed to examine the relationship between hospital surgical volume and 3-year survival for esophageal, biliary tract, and pancreatic cancer patients in Japan.

## METHODS

### Data source and subjects

Individual data on reported incident cases with follow-up information was retrieved from the Osaka Cancer Registry (OCR) database, a population-based cancer registry, which has been operating since December 1962 and covers the entire Osaka Prefecture, with a population of 8.8 million.^19^ Patient data from the OCR include sex, age at cancer diagnosis, vital status, and dates of death or the last follow-up for vital status. Tumor-specific data include cancer site, cancer stage, and date of cancer diagnosis. Treatment data include type of treatment (ie, surgery, chemotherapy, and radiation therapy), range of cancer resection, and hospital information at which patients were diagnosed and received treatment. Follow-up for vital status are routinely performed using death certificates. We identified incident cases of esophageal, biliary tract, and pancreatic cancers in the period 2006–2013, with follow-up and survival of more than 1 day. The loss to follow up was 0.4% to 0.7% for each cancer (eTable 1). Patients were followed up in December 2016 using official resident registries to verify vital status. Patients were treated in hospitals in Osaka, which are divided into eight medical referral regions, using relevant International Classification of Disease, 10^th^ Revision (ICD-10) diagnosis codes (esophageal: C15, biliary tract: C23–C24, and pancreatic: C25) for primary and multiple malignancies. The cancer stage at diagnosis was classified into the following three groups: 1) Localized: cancer is confined to the original organ: 2) Regional: cancer has spread to regional lymph nodes and/or to immediately adjacent tissues: 3) Distant: cancer has metastasized to distant organs.

**Figure 1.**
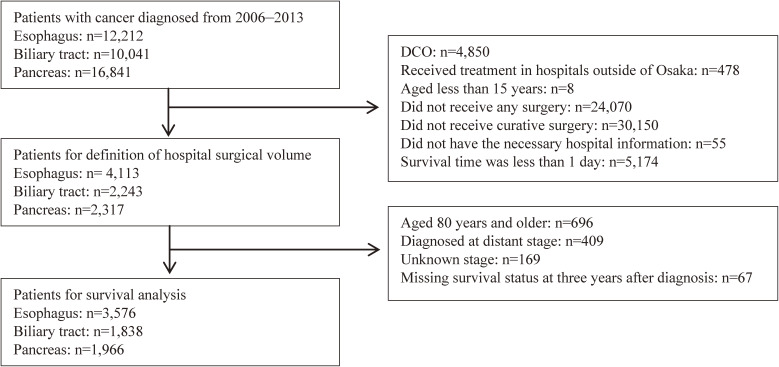
Flow chart for selection of patients in the Osaka Cancer Registry. DCO, death certification only.

Patients aged 15 through 79 undergoing curative surgery for these cancers from 2006 through 2013 were analyzed for survival rates. Patients with multiple cancers constituted 6.0% to 27.7% of patients by age and cancer site, so they were included to reduce bias due to observation period and age and improve the accuracy estimate (eTable 2).^20^^,^^21^ Patients whose survival status at 3 years after diagnosis was unknown, or whose cancer stage was distant or unknown, were excluded from the survival analyses (Figure 1).

The study was reviewed and approved by the Research Ethics Committee of the Osaka International Cancer Institute (no. 18-0018).

### Definition of hospital surgical volume

To define the hospital surgical volume, the average annual number of curative surgeries (ie, surgery, laparoscopic, and endoscopic treatment) through 2006 through 2013 for each of esophageal, biliary tract, and pancreatic cancer was calculated based on patients aged 15 years and older at all cancer stages (localized, regional, distant, unknown). Because curative surgery has been reported as the best option for long-term mortality in cancer patients,^22^ the number of curative surgeries was used as a measure for hospital surgical volume. Thus, using the average annual number of surgeries, tertile categories of hospital volume (high, middle, and low) were defined.

### Statistical analysis

The distribution of patient characteristics was assessed using Chi-squared tests for categorical variables. The Kaplan-Meier method and Cox proportional hazard model were used to analyze survival, regardless of the cause of death. Cumulative survival for each cancer was estimated using the Kaplan-Meier method according to hospital surgical volume. Hazard ratios (HRs) and 95% confidence intervals (CIs) were calculated using Cox proportional hazard regression models for 3-year mortality adjusting for patient characteristics. Covariates were age (15–59, 60–64, 65–69, 70–74, 75–79 years), sex (men, women), stage (localized, regional), chemotherapy (given, not given/unknown), radiation therapy (given, not given/unknown), residence and medical referral regions (different, same) as an index of distance between residence and hospital,^23^ and year of diagnosis (2006–2008, 2009–2011, 2012–2013). Stratified analyses by cancer stage (localized, regional) were also conducted, as 3-year mortality might differ across cancer stages.^24^

Differences were considered as statistically significant if *P* values were less than 0.05 by two-side test. We used the statistical software package STATA release 14 (Stata Corporation, College Station, TX, USA) for data management and statistical analysis.

## RESULTS

Characteristics of patients with esophageal (*n* = 3,576), biliary tract (*n* = 1,838), and pancreatic cancers (*n* = 1,966) according to hospital volume category are shown in Table 1, Table 2, and Table 3, respectively. Hospital surgical volumes were defined as follows: for esophageal cancer, three high-volume hospitals with a range of 53.3 to 70.1 surgical cases per year, seven middle-volume with 11.1 to 49.3 cases, and 86 low-volume with 0.1 to 10.6 cases (Table 1); for biliary tract cancer, 10 high-volume hospitals with 7.4 to 18.0 surgical cases per year, 18 middle-volume with 3.8 to 7.3 cases, and 72 low-volume with 0.1 to 3.6 cases (Table 2); for pancreatic cancer, 5 high-volume hospitals with 13.8 to 28.4 surgical cases per year, 14 middle-volume with 4.1 to 12.8 cases, and 70 low-volume with 0.1 to 4.0 cases (Table 3).

**Table 1.** Characteristics of esophageal cancer patients undergoing curative surgery at high-, middle-, and low-volume hospitals, based on data from the 2006–2013 Osaka Cancer Registry Database (*n* = 3,576)

			Hospital surgical volume			

			High	Middle	Low	*P* value^a^
Esophagus				
	Number of patients		1,235	1,171	1,170	
	Hospital characteristics				
		Number of hospitals	3	7	86	
		Procedure volume (range, cases per year)	53.3–70.1	11.1–49.3	0.1–10.6	
	Patient characteristics, *n* (%)				
	Age					0.356
		15–59 years	267 (21.6)	243 (20.8)	234 (20.0)	
		60–64 years	258 (20.9)	232 (19.8)	224 (19.2)	
		65–69 years	321 (26.0)	301 (25.7)	309 (26.4)	
		70–74 years	267 (21.6)	248 (21.2)	246 (21.0)	
		75–79 years	122 (9.9)	147 (12.6)	157 (13.4)	
	Sex					0.665
		Male	1,021 (82.7)	982 (83.9)	981 (83.9)	
		Female	214 (17.3)	189 (16.1)	189 (16.2)	
	Year of diagnosis					<0.001
		2006–2008	330 (26.7)	367 (31.3)	344 (29.4)	
		2009–2011	505 (40.9)	456 (38.9)	390 (33.3)	
		2012–2013	400 (32.4)	348 (29.7)	436 (37.3)	
	Stage					<0.001
		Localized	699 (56.6)	512 (43.7)	600 (51.3)	
		Regional	536 (43.4)	659 (56.3)	570 (48.7)	
	Chemotherapy					<0.001
		Given	461 (37.3)	543 (46.4)	426 (36.4)	
		Not given/Unknown	774 (62.7)	628 (53.6)	744 (63.6)	
	Radiation therapy					<0.001
		Given	165 (13.4)	90 (7.7)	107 (9.2)	
		Not given/Unknown	1,070 (86.6)	1,081 (92.3)	1,063 (90.9)	
	Residence and medical referral regions					<0.001
		Different	789 (63.9)	315 (26.9)	214 (18.3)	
		Same	446 (36.1)	856 (73.1)	956 (81.7)	

^a^Chi-squared test.

**Table 2.** Characteristics of biliary tract cancer patients undergoing curative surgery at high-, middle-, and low-volume hospitals, based on data from the 2006–2013 Osaka Cancer Registry Database (*n* = 1,838)

			Hospital surgical volume			

			High	Middle	Low	*P* value^a^
Biliary tract				
	Number of patients		642	618	578	
	Hospital characteristics				
		Number of hospitals	10	18	72	
		Procedure volume (range, cases per year)	7.4–18.0	3.8–7.3	0.1–3.6	
	Patient characteristics, *n* (%)				
	Age					0.904
		15–59 years	94 (14.6)	96 (15.5)	78 (13.5)	
		60–64 years	104 (16.2)	93 (15.1)	95 (16.4)	
		65–69 years	136 (21.2)	125 (20.2)	116 (20.1)	
		70–74 years	168 (26.2)	154 (24.9)	143 (24.7)	
		75–79 years	140 (21.8)	150 (24.3)	146 (25.3)	
	Sex					0.746
		Male	379 (59.0)	375 (60.7)	339 (58.7)	
		Female	263 (41.0)	243 (39.3)	239 (41.4)	
	Year of diagnosis					0.074
		2006–2008	187 (29.1)	202 (32.7)	169 (29.2)	
		2009–2011	256 (39.9)	238 (38.5)	202 (35.0)	
		2012–2013	199 (31.0)	178 (28.8)	207 (35.8)	
	Stage					0.057
		Localized	182 (28.4)	178 (28.8)	197 (34.1)	
		Regional	460 (71.7)	440 (71.2)	381 (65.9)	
	Chemotherapy					0.002
		Given	229 (35.7)	182 (29.5)	153 (26.5)	
		Not given/Unknown	413 (64.3)	436 (70.6)	425 (73.5)	
	Radiation therapy					0.007
		Given	18 (2.8)	7 (1.1)	4 (0.7)	
		Not given/Unknown	624 (97.2)	611 (98.9)	574 (99.3)	
	Residence and medical referral regions					<0.001
		Different	180 (28.0)	104 (16.8)	93 (16.1)	
		Same	462 (72.0)	514 (83.2)	485 (83.9)	

^a^Chi-squared test.

**Table 3.** Characteristics of pancreatic cancer patients undergoing curative surgery at high-, middle-, and low-volume hospitals, based on data from the 2006–2013 Osaka Cancer Registry Database (*n* = 1,966)

			Hospital surgical volume			

			High	Middle	Low	*P* value^a^
Pancreas				
	Number of patients		684	666	616	
	Hospital characteristics				
		Number of Hospitals	5	14	70	
		Procedure volume (range, cases per year)	13.8–28.4	4.1–12.8	0.1–4.0	
	Patient characteristics, *n* (%)				
	Age					0.014
		15–59 years	138 (20.2)	105 (15.8)	101 (16.4)	
		60–64 years	120 (17.5)	97 (14.6)	110 (17.9)	
		65–69 years	150 (21.9)	152 (22.8)	123 (20.0)	
		70–74 years	173 (25.3)	161 (24.2)	159 (25.8)	
		75–79 years	103 (15.1)	151 (22.7)	123 (20.0)	
	Sex					0.526
		Male	388 (56.7)	383 (57.5)	368 (59.7)	
		Female	296 (43.3)	283 (42.5)	248 (40.3)	
	Year of diagnosis					0.858
		2006–2008	205 (30.0)	196 (29.4)	176 (28.6)	
		2009–2011	253 (37.0)	234 (35.1)	221 (35.9)	
		2012–2013	226 (33.0)	236 (35.4)	219 (35.6)	
	Stage					0.005
		Localized	141 (20.6)	123 (18.5)	159 (25.8)	
		Regional	543 (79.4)	543 (81.5)	457 (74.2)	
	Chemotherapy					0.053
		Given	426 (62.3)	391 (58.7)	343 (55.7)	
		Not given/Unknown	258 (37.7)	275 (41.3)	273 (44.3)	
	Radiation therapy					<0.001
		Given	222 (32.5)	29 (4.4)	13 (2.1)	
		Not given/Unknown	462 (67.5)	637 (95.7)	603 (97.9)	
	Residence and medical referral regions					<0.001
		Different	322 (47.1)	149 (22.4)	98 (15.9)	
		Same	362 (52.9)	517 (77.6)	518 (84.1)	

^a^Chi-squared test.

Cancer stage differed by hospital surgical volume. The proportion of localized stage cancer was calculated for each of the three sites as follows: esophageal 56.6%, 43.7%, and 51.3% in high-, middle-, and low-volume hospitals, respectively; biliary tract, 28.4%, 28.8%, and 34.1%, respectively; pancreatic, 20.6%, 18.5%, and 25.8%, respectively. Patients more often go to hospitals in their home region in low-volume hospitals. By cancer site, the proportion of patients going to hospitals in their home region was as follows: esophageal, 36.1%, 73.1%, and 81.7% in high-, middle-, and low-volume hospitals, respectively; biliary tract, 72.0%, 83.2%, and 83.9%, respectively; pancreatic, 52.9%, 77.6%, and 84.1%, respectively.

**Figure 2.**
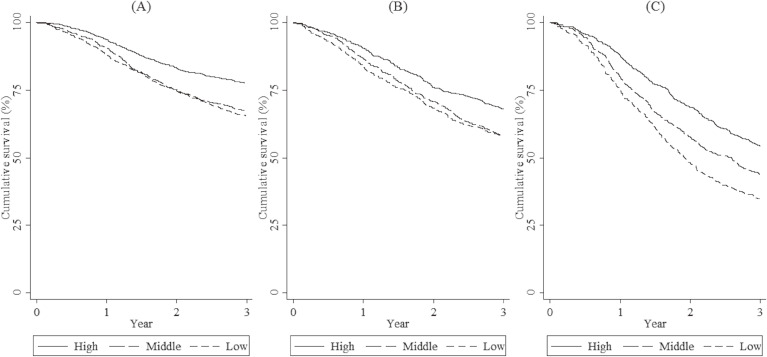
Kaplan-Meier estimates for 3-year survival among patients who received curative surgery for cancer at high-, middle-, and low-volume hospitals. Cancer types included are: esophagus (A), biliary tract (B), and pancreas (C).

Figure 2 shows 3-year cumulative survival curves of patients with esophageal, biliary tract, and pancreatic cancers diagnosed from 2006 through 2013 by hospital surgical volume group. Three-year survival was lower with decreasing hospital surgical volume for each site: esophageal, 77.6%, 67.3%, and 65.5% in high-, middle- and low-volume hospitals, respectively; biliary tract, 67.9%, 58.3%, and 58.0%, respectively; pancreatic, 54.2%, 43.7% and 34.7%, respectively.

Table 4 shows HRs for 3-year mortality by hospital surgical volume. After adjustment for patient characteristics using Cox regression models, significantly higher 3-year mortality was found in middle- and low-volume hospitals compared to high-volume hospitals. HRs for esophageal cancer were 1.34 (95% CI, 1.14–1.58) in middle-volume and 1.57 (95% CI, 1.33–1.86) in low-volume hospitals; for biliary tract cancer, HRs were 1.39 (95% CI, 1.15–1.67) in middle-volume and 1.57 (95% CI, 1.30–1.89) in low-volume hospitals; and for pancreatic cancer, HRs were 1.38 (95% CI, 1.16–1.63) in middle-volume and 1.90 (95% CI, 1.60–2.25) in low-volume hospitals. HRs for covariate variables by site are shown as supplementary data in eTable 3.

**Table 4.** Association between hospital surgical volume and 3-year mortality in esophageal, biliary tract, and pancreatic cancer patients who received curative surgery, with and without adjustment for patient characteristics

Site	Hospital surgical volume	Crude HR (95% CI)	Adjusted HR^a^ (95% CI)
Esophagus			
	High	1 (reference)	1 (reference)
	Middle	1.56 (1.34–1.82)	1.34 (1.14–1.58)
	Low	1.67 (1.43–1.95)	1.57 (1.33–1.86)
Biliary tract			
	High	1 (reference)	1 (reference)
	Middle	1.39 (1.16–1.67)	1.39 (1.15–1.67)
	Low	1.44 (1.19–1.73)	1.57 (1.30–1.89)
Pancreas			
	High	1 (reference)	1 (reference)
	Middle	1.39 (1.19–1.61)	1.38 (1.16–1.63)
	Low	1.79 (1.54–2.08)	1.90 (1.60–2.25)

CI, confidence interval; HR, hazard ratio.

^a^Adjusted for age, sex, year of diagnosis, stage, chemotherapy, radiation therapy, and residence and medical referral regions.

The results of HRs for hospital surgical volumes stratified by cancer stage are shown in Table 5. HRs for localized cases, were higher (range, 1.27 to 1.56) in middle-volume than high-volume hospitals, though the HRs of middle-volume hospitals were not significant in biliary tract and pancreatic cancers. In low-volume hospitals, HRs were higher (range, 1.30 to 2.66) than high-volume hospitals, and the range was wider than that in middle-volume hospitals, though the HRs of low-volume hospitals were not significant in biliary tract cancer.

**Table 5.** Association between hospital surgical volume and 3-year mortality in esophageal, biliary tract, and pancreatic cancer patients who received curative surgery, with and without adjustment for patient characteristics, classifying by cancer stage

Cancer Stage		Localized		Regional	

Site	Hospital surgical volume	Crude HR (95% CI)	Adjusted HR^a^ (95% CI)	Crude HR (95% CI)	Adjusted HR^a^ (95% CI)
Esophagus					
	High	1 (reference)	1 (reference)	1 (reference)	1 (reference)
	Middle	1.59 (1.19–2.12)	1.36 (1.00–1.85)	1.30 (1.08–1.56)	1.33 (1.09–1.62)
	Low	1.65 (1.25–2.18)	1.47 (1.09–1.98)	1.59 (1.32–1.91)	1.60 (1.30–1.96)
Biliary tract					
	High	1 (reference)	1 (reference)	1 (reference)	1 (reference)
	Middle	1.36 (0.81–2.28)	1.27 (0.75–2.15)	1.43 (1.18–1.74)	1.40 (1.15–1.70)
	Low	1.50 (0.91–2.47)	1.30 (0.77–2.17)	1.57 (1.28–1.92)	1.58 (1.29–1.93)
Pancreas					
	High	1 (reference)	1 (reference)	1 (reference)	1 (reference)
	Middle	1.59 (0.95–2.65)	1.56 (0.90–2.69)	1.35 (1.15–1.58)	1.35 (1.13–1.61)
	Low	2.78 (1.77–4.38)	2.66 (1.61–4.38)	1.82 (1.55–2.13)	1.78 (1.49–2.14)

CI, confidence interval; HR, hazard ratio.

^a^Adjusted for age, sex, year of diagnosis, chemotherapy, radiation therapy, and residence and medical referral regions.

HRs for regional cases were significantly higher (range, 1.33 to 1.40) in middle-volume than high-volume hospitals. HRs in low-volume hospitals were also significantly higher (range, 1.58 to 1.78) than high-volume hospitals.

## DISCUSSION

Increased hospital surgical volume related to lower 3-year mortality for esophageal, biliary tract, and pancreatic cancers in Osaka, after adjusting for patient characteristics. Our findings were consistent with previous studies in terms of larger risk for long-term mortality in low-volume than high-volume hospitals for these three cancers.^4^^,^^6^^,^^8^^,^^18^^,^^25^ Our results suggest that 3-year survival for patients with these three cancers might be improved by having curative surgery in high-volume hospitals, rather than middle- and low-volume hospitals in Osaka, Japan.

In this study, 3-year mortality was significantly different between high- and middle-volume hospitals for esophageal, biliary tract, and pancreatic cancer. Compared with middle-volume hospitals, high-volume hospitals demonstrated superior impact on patient survival. Looking at our results and those of previous studies, it appears that this tendency differs by cancer site because prevalence and level of technical-medical expertise differ by cancer site. For example, patients treated for stomach cancer, which has high prevalence in Japan, in middle-volume hospitals had a similar mortality risk to those treated in high-volume hospitals.^16^^,^^17^ Another previous study for rectal cancer, which has high prevalence in Japan, found that survival of patients who had surgery in high-volume hospitals did not largely differ from that in middle- and low-volume hospitals.^26^ Our findings suggest that patients who need curative surgery for esophageal, biliary tract, and pancreatic cancer, which require advanced technical-medical skills, will benefit from attending high-volume rather than middle-volume hospitals.

The impact of hospital surgical volume on 3-year mortality might differ by site and cancer stage. For esophageal cancer, hospital surgical volume was significantly associated with 3-year mortality, and the associations between hospital surgical volume and 3-year mortality did not differ by cancer stage.

For localized biliary tract cancer, point estimates of HRs of middle- and low-volume hospitals to high-volume hospital were high, though they were not significant. On the other hand, for regional cases, point estimates of HRs of middle- and low-volume hospitals were significantly higher than high-volume hospitals, and higher than those for localized cases. This difference may be due to treatment difficulties, such as additional resection for invasive biliary tract cancer patients.^27^ Their surgeries may require higher level technical skills than localized cases, resulting in the difference in HRs by hospital surgical volume, especially in regional cases.

For pancreatic cancer, the point estimate of HR of low-volume hospitals was higher for localized than regional stage cases, although the 95% CIs overlapped in these cases. A previous study has reported that localized pancreatic cancer patients who received pancreaticoduodenectomy at low-volume hospitals are more likely to have margin-positive resections, and their long-term mortality is higher.^28^ Our results agree with this study and suggest that receiving curative surgery in low-volume hospitals is risky, especially for localized pancreatic cancer patients. The environment of hospitals providing combined modality therapy might be associated with high mortality in low-volume hospitals, because access to medical equipment for radiation therapy seems better in high-volume hospitals than in low-volume hospitals. However, we could not determine the hospital environment from the OCR database. In this study, for localized pancreatic cancer patients, the frequency of chemotherapy was higher in low-volume hospitals than in high-volume hospitals (36.5% vs 22.0%) and that of radiation therapy was low in both low- and high-volume hospitals (0.0% vs 4.3%) (eTable 4). Moreover, the adjusted HR of chemotherapy was 0.82 (95% CI, 0.56–1.22) and that of radiation therapy was 0.57 (95% CI, 0.16–2.04); both HRs were not significant (eTable 5). These results may suggest that the high mortality in low-volume hospitals for localized pancreatic cancer patients is not associated with combined modality therapy.

Distance between residence and hospital was not associated with improved 3-year mortality for esophageal, biliary tract, and pancreatic cancer patients. Previous studies have reported that the impact of travel distance is mediated through hospital volume for biliary tract and pancreatic cancer.^18^^,^^29^ Although the distance was not defined using specific numerical values in our study, our results support these studies and suggest that the impact of hospital surgical volume on 3-year mortality is stronger than travel distance for esophageal, biliary tract, and pancreatic cancer patients.

This study has several limitations. First, selection bias may have occurred in this study, thus our results need to be interpreted carefully. We described the number and proportion, including patients who were excluded from the analysis (eTable 1); however, the proportion of patients who were excluded was relatively small. Furthermore, to examine the degree of the selection bias, we calculated HRs for 3-year mortality by hospital surgical volume, including patients aged 80 years and older and/or with unknown stage (eTable 6). The results did not differ from our main results (Table 4). Therefore, the effect of excluding patients aged 80 years and older and/or with unknown stage may be small. Second, we did not take into account other hospital characteristics, such as the number of medical staff.^30^ Furthermore, we could not include some covariates, such as comorbidity and socioeconomic position. Finally, the present study is not representative of the general population in Japan because we only used data from one part of the country, Osaka.

In conclusion, the results suggest that 3-year mortality is significantly lower at high-volume hospitals compared with middle- and low-volume ones for esophageal, biliary tract, and pancreatic cancers. Our study suggests that the prognosis for patients who require curative surgery for esophageal, biliary tract, and pancreatic cancer may be improved by referral to high-volume hospitals.

## APPENDIX A. SUPPLEMENTARY DATA

eTable 1. Characteristics of all patients with esophageal, biliary tract, and pancreatic cancer at high-, middle-, and low-volume hospitals, based on data from the 2006–2013 Osaka Cancer Registry Database

eTable 2. Prevalence of patients with primary and multiple cancers for esophageal, biliary tract, and pancreatic cancers by age

eTable 3. Crude and adjusted hazard ratios for covariates by cancer site

eTable 4. Treatment of patients with localized pancreatic cancer by hospital surgical volume

eTable 5. Association between adjuvant therapy and 3-year mortality in patients with localized pancreatic cancer

eTable 6. Association between hospital surgical volume and 3-year mortality in esophageal, biliary tract, and pancreatic cancer patients who received curative surgery, with and without adjustment for patient characteristics
