# Supplementary material for: Hospital Surgical Volume and 3-Year Mortality in Severe Prognosis Cancers: A Population-Based Study Using Cancer Registry Data
Source: J Epidemiol. 2021 Jan 5;31(1):52–8. doi: 10.2188/jea.JE20190242 (PMC7738649; doi:10.2188/jea.JE20190242)
Supplement: Supplementary file 1 [file je-31-052-s001.pdf]

eTable 1. Characteristics of all patients with esophageal, biliary tract, and pancreatic cancer at high-, middle-, and low-volume hospitals, based on data from the 2006–2013 Osaka Cancer Registry Database

		Esophagus				Biliary tract				Pancreas			
		Hospital surgical volume				Hospital surgical volume				Hospital surgical volume			
		High	Middle	Low	All[a]	High	Middle	Low	All[a]	High	Middle	Low	All[a]
Number of patients		2,684	2,365	3,605	12,212	1,603	1,698	2,027	10,041	2,354	2,545	3,698	16,841
DCO[b], n (%)		3 (0.1)	10 (0.4)	70 (1.9)	905 (7.4)	10 (0.6)	16 (0.9)	170 (8.4)	1,601 (15.9)	3 (0.1)	25 (0.9)	185 (5.0)	2,344 (13.9)
Age, n (%)													
	0–14 years	0 (0.0)	0 (0.0)	0 (0.0)	0 (0.0)	0 (0.0)	0 (0.0)	0 (0.0)	0 (0.0)	0 (0.0)	2 (0.1)	0 (0.0)	8 (0.1)
	15–59 years	551 (20.5)	458 (19.4)	602 (16.7)	2,056 (16.8)	202 (12.6)	189 (11.1)	178 (8.8)	804 (8.0)	484 (20.6)	379 (14.9)	514 (13.9)	2,026 (12.0)
	60–64 years	532 (19.8)	425 (18.0)	610 (16.9)	2,090 (17.1)	212 (13.2)	197 (11.6)	212 (10.5)	869 (8.7)	392 (16.7)	349 (13.7)	525 (14.2)	1,959 (11.6)
	65–69 years	625 (23.3)	545 (23.0)	804 (22.3)	2,592 (21.2)	261 (16.3)	287 (16.9)	304 (15.0)	1,236 (12.3)	486 (20.7)	472 (18.6)	672 (18.2)	2,580 (15.3)
	70–74 years	531 (19.8)	472 (20.0)	731 (20.3)	2,362 (19.3)	358 (22.3)	335 (19.7)	395 (19.5)	1,647 (16.4)	472 (20.1)	557 (21.9)	733 (19.8)	2,996 (17.8)
	75–79 years	292 (10.9)	298 (12.6)	475 (13.2)	1,632 (13.4)	309 (19.3)	362 (21.3)	422 (20.8)	1,849 (18.4)	333 (14.2)	467 (18.4)	673 (18.2)	2,918 (17.3)
	≥80 years	153 (5.7)	167 (7.1)	383 (10.6)	1,480 (12.1)	261 (16.3)	328 (19.3)	516 (25.5)	3,636 (36.2)	187 (7.9)	319 (12.5)	581 (15.7)	4,354 (25.9)
	Missing	0 (0.0)	0 (0.0)	0 (0.0)	0 (0.0)	0 (0.0)	0 (0.0)	0 (0.0)	0 (0.0)	0 (0.0)	0 (0.0)	0 (0.0)	0 (0.0)
Stage, n (%)													
	Localized	1,006 (37.5)	737 (31.2)	1,085 (30.1)	3,306 (27.1)	266 (16.6)	282 (16.6)	369 (18.2)	1,165 (11.6)	182 (7.7)	193 (7.6)	311 (8.4)	986 (5.9)
	Regional	1,078 (40.2)	1,137 (48.1)	1,594 (44.2)	4,606 (37.7)	885 (55.2)	912 (53.7)	975 (48.1)	3,543 (35.3)	1,122 (47.7)	1,167 (45.9)	1,510 (40.8)	5,019 (29.8)
	Distant	362 (13.5)	442 (18.7)	735 (20.4)	2,181 (17.9)	352 (22.0)	403 (23.7)	499 (24.6)	2,317 (23.1)	855 (36.3)	1,122 (44.1)	1,651 (44.7)	6,602 (39.2)
	Unknown	238 (8.9)	49 (2.1)	191 (5.3)	1,181 (9.7)	100 (6.2)	101 (6.0)	184 (9.1)	1,396 (13.9)	195 (8.3)	63 (2.5)	226 (6.1)	1,852 (11.0)
	Missing	0 (0.0)	0 (0.0)	0 (0.0)	938 (7.7)	0 (0.0)	0 (0.0)	0 (0.0)	1,620 (16.1)	0 (0.0)	0 (0.0)	0 (0.0)	2,382 (14.1)
Surgery, n (%)													
Given													
	Curative	1,434 (53.4)	1,355 (57.3)	1,328 (36.8)	4,209 (34.5)	761 (47.5)	754 (44.4)	730 (36.0)	2,323 (23.1)	797 (33.9)	777 (30.5)	740 (20.0)	2,412 (14.3)
	Palliative	335 (12.5)	235 (9.9)	424 (11.8)	1,048 (8.6)	534 (33.3)	571 (33.6)	696 (34.3)	1,893 (18.9)	419 (17.8)	681 (26.8)	1,006 (27.2)	2,319 (13.8)

		Unknown	25 (0.9)	18 (0.8)	212 (5.9)	276 (2.3)	9 (0.6)	33 (1.9)	200 (9.9)	260 (2.6)	16 (0.7)	38 (1.5)	201 (5.4)	284 (1.7)
	Not given		866 (32.3)	756 (32.0)	1,535 (42.6)	5,105 (41.8)	285 (17.8)	326 (19.2)	375 (18.5)	3,306 (32.9)	1,111 (47.2)	1,029 (40.4)	1,654 (44.7)	8,349 (49.6)
	Unknown		24 (0.9)	1 (0.0)	106 (2.9)	636 (5.2)	14 (0.9)	14 (0.8)	26 (1.3)	639 (6.4)	11 (0.5)	20 (0.8)	97 (2.6)	1,095 (6.5)
	Missing		0 (0.0)	0 (0.0)	0 (0.0)	938 (7.7)	0 (0.0)	0 (0.0)	0 (0.0)	1,620 (16.1)	0 (0.0)	0 (0.0)	0 (0.0)	2,382 (14.1)
Follow-up status, n (%)														
		Missing	20 (0.7)	13 (0.5)	32 (0.9)	88 (0.7)	8 (0.5)	12 (0.7)	8 (0.4)	45 (0.4)	11 (0.5)	9 (0.4)	23 (0.6)	72 (0.4)

DCO, death certification only.

[a]All category included patients treated in hospitals which were not included in any surgical volume category.

[b]DCO cases were classified into surgical volume category, considering the reported hospitals as the treatment hospitals.

eTable 2. Prevalence of patients with primary and multiple cancers for esophageal, biliary tract, and pancreatic cancers by age

	Esophagus		Biliary tract		Pancreas	
Age	Primary N (%)	Multiple N (%)	Primary N (%)	Multiple N (%)	Primary N (%)	Multiple N (%)
15–59 years	615 (82.7)	129 (17.3)	252 (94.0)	16 (6.0)	322 (93.6)	22 (6.4)
60–64 years	579 (81.1)	135 (18.9)	255 (87.3)	37 (12.7)	285 (87.2)	42 (12.8)
65–69 years	741 (79.6)	190 (20.4)	322 (85.4)	55 (14.6)	366 (86.1)	59 (13.9)
70–74 years	569 (74.8)	192 (25.2)	385 (82.8)	80 (17.2)	398 (80.7)	95 (19.3)
75–79 years	308 (72.3)	118 (27.7)	347 (79.6)	89 (20.4)	307 (81.4)	70 (18.6)
15–79 years	2,812 (78.6)	764 (21.4)	1,561 (84.9)	277 (15.1)	1,678 (85.4)	288 (14.7)

eTable 3. Crude and adjusted hazard ratios for covariates by cancer site

		Esophagus		Biliary tract		Pancreas	
		Crude HR (95% CI)	Adjusted HR[a] (95% CI)	Crude HR (95% CI)	Adjusted HR[a] (95% CI)	Crude HR (95% CI)	Adjusted HR[a] (95% CI)
Age							
	15–59 years	1 (reference)	1 (reference)	1 (reference)	1 (reference)	1 (reference)	1 (reference)
	60–64 years	0.99 (0.81–1.20)	1.02 (0.84–1.25)	1.27 (0.95–1.69)	1.21 (0.90–1.62)	1.30 (1.06–1.59)	1.30 (1.06–1.60)
	65–69 years	1.09 (0.91–1.31)	1.09 (0.91–1.30)	1.45 (1.11–1.90)	1.32 (1.01–1.73)	1.11 (0.91–1.35)	1.14 (0.93–1.39)
	70–74 years	1.13 (0.93–1.36)	1.27 (1.05–1.53)	1.45 (1.11–1.88)	1.41 (1.08–1.83)	1.16 (0.95–1.40)	1.24 (1.02–1.51)
	75–79 years	1.54 (1.25–1.89)	1.81 (1.47–2.24)	1.55 (1.19–2.01)	1.53 (1.17–2.00)	1.53 (1.25–1.86)	1.53 (1.25–1.87)
Sex							
	Male	1 (reference)	1 (reference)	1 (reference)	1 (reference)	1 (reference)	1 (reference)
	Female	0.64 (0.53–0.77)	0.64 (0.53–0.77)	0.80 (0.69–0.93)	0.78 (0.67–0.91)	0.76 (0.68–0.86)	0.78 (0.69–0.89)
Year of diagnosis							
	2006–2008	1 (reference)	1 (reference)	1 (reference)	1 (reference)	1 (reference)	1 (reference)
	2009–2011	0.87 (0.75–1.01)	0.95 (0.82–1.10)	0.90 (0.76–1.07)	0.86 (0.72–1.02)	0.87 (0.75–1.00)	0.86 (0.74–0.99)
	2012–2013	0.82 (0.70–0.95)	0.84 (0.72–0.98)	0.74 (0.61–0.89)	0.68 (0.56–0.82)	0.68 (0.58–0.79)	0.64 (0.55–0.75)
Stage							
	Localized	1 (reference)	1 (reference)	1 (reference)	1 (reference)	1 (reference)	1 (reference)
	Regional	3.11 (2.72–3.55)	3.02 (2.59–3.53)	3.36 (2.71–4.16)	3.28 (2.62–4.09)	2.79 (2.32–3.36)	3.10 (2.56–3.76)
Chemotherapy							
	Given	1 (reference)	1 (reference)	1 (reference)	1 (reference)	1 (reference)	1 (reference)
	Not given / Unknown	0.53 (0.47–0.60)	0.98 (0.84–1.13)	0.67 (0.58–0.78)	0.85 (0.72–1.00)	0.97 (0.86–1.10)	1.18 (1.03–1.35)
Radiation therapy							
	Given	1 (reference)	1 (reference)	1 (reference)	1 (reference)	1 (reference)	1 (reference)
	Not given / Unknown	0.60 (0.51–0.71)	0.65 (0.54–0.78)	0.91 (0.51–1.61)	1.03 (0.58–1.84)	1.13 (0.95–1.35)	0.91 (0.74–1.13)
Residence and medical referral regions							
	Different	1 (reference)	1 (reference)	1 (reference)	1 (reference)	1 (reference)	1 (reference)
	Same	1.32 (1.16–1.51)	1.09 (0.95–1.26)	1.31 (1.08–1.59)	1.16 (0.95–1.41)	1.26 (1.10–1.45)	1.04 (0.90–1.20)

CI, confidence interval; HR, hazard ratio.

[a]Adjusted for age, sex, year of diagnosis, stage, chemotherapy, radiation therapy, residence and medical referral regions, and hospital surgical volume.

eTable 4. Treatment of patients with localized pancreatic cancer by hospital surgical volume

		Hospital surgical volume			
		High<br>N (%)	Middle<br>N (%)	Low<br>N (%)	P value
Chemotherapy					0.023
	Given	31 (22.0)	36 (29.3)	58 (36.5)	
	Not given / Unknown	110 (78.0)	87 (70.7)	101 (63.5)	
Radiation therapy					0.011
	Given	6 (4.3)	1 (0.8)	0 (0.0)	
	Not given / Unknown	135 (95.7)	122 (99.2)	159 (100.0)	
Combined modality therapy					<0.001
	Curative surgery only	110 (78.0)	86 (69.9)	101 (63.5)	
	Curative surgery + Chemotherapy	25 (17.7)	36 (29.3)	58 (36.5)	
	Curative surgery + Radiation therapy	0 (0.0)	1 (0.8)	0 (0.0)	
	Curative surgery + Chemotherapy + Radiation therapy	6 (4.3)	0 (0.0)	0 (0.0)	

[a]Chi-squared test.

eTable 5. Association between adjuvant therapy and 3-year mortality in patients with localized pancreatic cancer

		Crude HR (95% CI)	Adjusted HR[a] (95% CI)
Chemotherapy			
	Given	1 (reference)	1 (reference)
	Not given / Unknown	0.72 (0.50–1.03)	0.82 (0.56–1.22)
Radiation therapy			
	Given	1 (reference)	1 (reference)
	Not given / Unknown	0.69 (0.22–2.16)	0.57 (0.16–2.04)

CI, confidence interval; HR, hazard ratio.

[a]Adjusted for age, sex, year of diagnosis, stage, chemotherapy, radiation therapy, residence and medical referral regions, and hospital surgical volume.

eTable 6. Association between hospital surgical volume and 3-year mortality in esophageal, biliary tract, and pancreatic cancer patients who received curative surgery, with and without adjustment for patient characteristics

Site	Hospital surgical volume	Crude HR (95% CI)	Adjusted HR[a] (95% CI)
Esophagus			
	High	1 (reference)	1 (reference)
	Middle	1.50 (1.29–1.73)	1.33 (1.14–1.56)
	Low	1.64 (1.42–1.89)	1.58 (1.35–1.85)
Biliary tract			
	High	1 (reference)	1 (reference)
	Middle	1.32 (1.11–1.56)	1.30 (1.10–1.54)
	Low	1.43 (1.21–1.69)	1.52 (1.28–1.81)
Pancreas			
	High	1 (reference)	1 (reference)
	Middle	1.35 (1.17–1.55)	1.33 (1.14–1.55)
	Low	1.72 (1.50–1.98)	1.80 (1.54–2.11)

CI, confidence interval; HR, hazard ratio.

[a]Adjusted for age, sex, year of diagnosis, stage, chemotherapy, radiation therapy, and residence and medical referral regions. For survival analysis, patients aged 80 years and older and/or with unknown stage were included.
